# Editorial: Insights in invertebrate physiology 2021

**DOI:** 10.3389/fphys.2023.1157932

**Published:** 2023-02-13

**Authors:** Sylvia Anton, Silke Sachse, Robert Huber

**Affiliations:** ^1^ IGEPP, INRAE, Institut Agro, University Rennes, CEDEX, Angers, France; ^2^ Research Group Olfactory Coding, Max Planck Institute for Chemical Ecology, Jena, Germany; ^3^ Bowling Green State University, Biological Sciences, Bowling Green, OH, United States

**Keywords:** insects, crustaceans, neurophysiology, neuromodulation, sensory systems

This Research Topic, part of a series of Research Topics in Frontiers in Physiology, aims to highlight new insights, novel developments, current challenges, latest discoveries, recent advances and future perspectives in the field of Invertebrate Physiology. It sheds light on the progress made in the past decade and indicates future challenges on invertebrate neurobiology, behavior and neuromodulation. The Research Topic includes two Original Research articles, three Reviews, one Mini Review, as well as a Perspective and one Hypothesis and Theory article. Five articles report on the neurobiology of insects, whereas one review takes a larger view on neuropeptides in different invertebrates. One Review and one perspective article focus on neuronal control of behavior in crayfish. The contributions reflect several major research fields covered by Frontiers in Invertebrate Physiology during the last decade, without being exhaustive.

A first group of articles, exploring the detection and processing of different sensory modalities in insects focuses on different levels of sensory pathways. Lizana et al. summarize in their review how advances in molecular approaches which led to the identification of various chemosensory proteins have allowed to better understand detection of behaviorally-relevant odors not only in model insects such as *Drosophila melanogaster*, *Bombyx mori* or *Tribolium castaneum*- but also in non-model species, including pest insects. They discuss the perspectives of these findings for the development of alternative pest management strategies. Bimodal integration of olfactory and mechanosensory information in form of wind speed in the primary olfactory integration center, the insect antennal lobe is explored with a modeling approach, based on experimental data by Patel et al. The authors discuss the potential relevance of their findings with regard to behavioral approach of an insect towards an odor source and postulate that odor plume tracking and odor discrimination are performed in a context-dependent manner. Higher order processing of sensory information is investigated by Schmalz et al. with a focus on vision. Based on multi-unit extracellular recordings, the authors describe how different behaviorally-relevant parameters of visual stimuli, such as brightness and wavelength are categorized within specific subpopulations of mushroom body output neurons in the honeybee. Switching to internal, rather than external sensing, Ratnaparkhi and Sudhakaran review how information on nutrient sensing within the fat body of the fruitfly is relayed to the brain and finally acts on the modulation of synthesis and release of insulin-like peptides, which are involved in the regulation of metabolism.

Two other papers focus on the function of neural circuits involved in locomotor behavior and its modulation. Rana et al. use an electrophysiological approach to investigate the modulation of nociceptive behavior by a parasitoid venom. The authors provide evidence that nociceptive stimuli are still transmitted to the brain in cockroaches stung and injected with venom by a parasitoid wasp, but that neurons within the locomotor control center, the central complex, reduce their firing duration in response to noxious stimuli, thus potentially explaining the lack of defensive behavior. Herberholz reviews what is known about the giant escape neurons in crayfish, with a comprehensive historical overview over important findings accumulated during the last almost 100 years. More specifically, recent physiological findings on freely behaving crayfish, and modulation of escape behavior and its underlying neuronal mechanisms, are summarized and future challenges are described. In the perspectives part of this review, Herberholz joins the ideas put forward by Stein et al. in their perspectives article. Here, new avenues to investigate neurophysiological mechanisms in crustaceans, by using genetic and molecular tools in a parthenogenetically reproducing crayfish, *Procambarus virginalis* are suggested to fill knowledge gaps in otherwise well-investigated crustacean nervous systems.

A review by Wegener and Chen on the distribution and neuromodulatory functions of Allatostatin A (AstA) signaling in various invertebrates provides an overview on the evolution of these peptides and their receptors across phyla and summarizes non-allatostatic functions. Whereas AstA has been shown to be an important neuromodulating peptide involved in many functions, such metabolism, food intake, growth, but also locomotion, sleep or learning, new functions are still to be discovered. Evolutionarily, AstA signalling in the modulation of gut movement, heartbeat and feeding/foraging appears to be conserved in Panarthropoda and Ecdysozoa. Future challenges include the characterization of ligand-receptor relationships and of AstA physiological function in a representative diversity of model species.

The Research Topic of articles in this Research Topic highlights some important knowledge gaps in the field of invertebrate neurophysiology. With modern tools becoming available in non-model insects, perspectives arise to investigate cellular and molecular mechanisms in peripheral and central neuronal functions as well as neuromodulation. A comparative approach across invertebrate taxa adds valuable new insights to existing knowledge from model insects.

